# Role of adenosine in the antiepileptic effects of deep brain stimulation

**DOI:** 10.3389/fncel.2014.00312

**Published:** 2014-10-02

**Authors:** Maisa F. Miranda, Clement Hamani, Antônio-Carlos G. de Almeida, Beatriz O. Amorim, Carlos E. Macedo, Maria José S. Fernandes, José N. Nobrega, Mayra C. Aarão, Ana Paula Madureira, Antônio M. Rodrigues, Monica L. Andersen, Sergio Tufik, Luiz E. Mello, Luciene Covolan

**Affiliations:** ^1^Laboratório de Neurociência Experimental e Computacional, Universidade Federal de São João del-ReiSão João del-Rei, Brazil; ^2^Disciplina de Neurofisiologia, Universidade Federal de São PauloSão Paulo, Brazil; ^3^Behavioural Neurobiology Laboratory, Centre for Addiction and Mental HealthToronto, Canada; ^4^Division of Neurosurgery, Toronto Western Hospital, University of TorontoToronto, Canada; ^5^Departamento de Psicobiologia, Universidade Federal de São PauloSão Paulo, Brazil; ^6^Disciplina de Neurologia Experimental, Universidade Federal de São PauloSão Paulo, Brazil

**Keywords:** deep brain stimulation, epilepsy, thalamus, anterior nucleus, seizures, adenosine

## Abstract

Despite the effectiveness of anterior thalamic nucleus (AN) deep brain stimulation (DBS) for the treatment of epilepsy, mechanisms responsible for the antiepileptic effects of this therapy remain elusive. As adenosine modulates neuronal excitability and seizure activity in animal models, we hypothesized that this nucleoside could be one of the substrates involved in the effects of AN DBS. We applied 5 days of stimulation to rats rendered chronically epileptic by pilocarpine injections and recorded epileptiform activity in hippocampal slices. We found that slices from animals given DBS had reduced hippocampal excitability and were less susceptible to develop ictal activity. In live animals, AN DBS significantly increased adenosine levels in the hippocampus as measured by microdialysis. The reduced excitability of DBS *in vitro* was completely abolished in animals pre-treated with A1 receptor antagonists and was strongly potentiated by A1 receptor agonists. We conclude that some of the antiepileptic effects of DBS may be mediated by adenosine.

## Introduction

Deep brain stimulation (DBS) involves the delivery of current to the brain parenchyma though implanted electrodes. Over the last decade, preclinical and clinical studies have shown that DBS applied to the anterior thalamic nucleus (AN) reduces seizure rate and increases the latency for the development of seizures and status epilepticus (SE; Mirski et al., 1997; Hodaie et al., 2002; Hamani et al., 2004, 2008; Kerrigan et al., 2004; Andrade et al., 2006; Takebayashi et al., 2007a,b; Fisher et al., 2010). Despite the efficacy of AN DBS, mechanisms involved in the antiepileptic effects of this therapy remain elusive.

We have recently shown that AN DBS reduced the frequency of spontaneous recurrent seizures in chronic epileptic animals (Covolan et al., 2014). In addition, animals receiving stimulation had a significant decrease in hippocampal excitability. This proved to be a more reliable measure of the effectiveness of DBS than frequency of seizures, due to the variability in seizure rate that occurs not only across animals but also in the same animals over time (Covolan et al., 2014).

Adenosine and other purines are known modulators of neuronal excitability and seizure activity in experimental animals and humans (Dunwiddie, 1980; Turski et al., 1985; O’Brien, 1988; Arvin et al., 1989; During and Spencer, 1992; Boison et al., 1999; Huber et al., 2001; Zuchora et al., 2001; Anschel et al., 2004; Boison, 2005, 2006, 2008, 2009; Cunha, 2005; Vianna et al., 2005; Pagonopoulou et al., 2006; Li et al., 2007, 2011; Boison and Stewart, 2009; Van Dycke et al., 2010, 2011; Hargus et al., 2012). In rats, adenosine injections directly onto the epileptic focus (Anschel et al., 2004) or the hippocampus improve seizure control (Van Dycke et al., 2010). Synthetic polymers capable of releasing adenosine into the ventricle have also been shown to reduce seizure activity (Boison et al., 1999). In preclinical models, SE up-regulates adenosine kinase (ADK), a key metabolic enzyme for the regulation of extracellular adenosine (Aronica et al., 2011). Fibroblasts engineered to release adenosine by inactivating ADK and adenosine deaminase grafted into the ventricles of kindled rats protected the animals from behavioral seizures and afterdischarges (Huber et al., 2001).

In thalamic slices from ferrets, stimulation-induced glutamate and adenosine release abolished spontaneous spindle oscillations (Tawfik et al., 2010). In slices from mice, thalamic stimulation increased ATP release and induced an accumulation in extracellular adenosine (Bekar et al., 2008). In mice, A1 receptor activation depressed excitatory transmission *in vitro* and reduced both tremor- and DBS-induced side effects *in vivo* (Bekar et al., 2008).

Bearing in mind the fact that adenosine modulates seizures in animal models and is involved in mechanisms of thalamic DBS for tremor (Bekar et al., 2008), we hypothesized that this nucleoside could be one of the substrates involved in the antiepileptic effects of AN DBS.

## Results

### AN-DBS induces adenosine release in hippocampus

**Figure 1:**
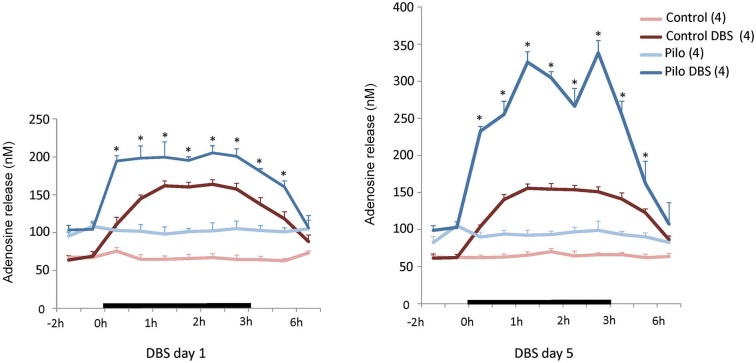
**Anterior thalamic nucleus DBS effects on hippocampal adenosine release**. On the first day of stimulation (left panel), a significant increase in adenosine release was measured soon after DBS was started, persisting for almost 2 h after stimulation was discontinued. On the fifth day of DBS (right panel) a similar trend was seen but adenosine levels in Pilo + DBS animals were almost twice as high as those recorded on stimulation day 1. Values are means and SEM. *N* = 4 for all groups. *p < 0.012 (day 1) and *p* < 0.0062 (day 5) Pilo + DBS compared to Pilo + Sham, Bonferroni-corrected tests. Other significant comparisons are omitted for the sake of clarity.

As shown in Figure 1, AN DBS increased hippocampal levels of adenosine in both epileptic and non-epileptic animals (DBS main effect *F*_(1,12)_ = 527.59, *p* < 0.0001). This increase was significantly higher in Pilo + DBS rats compared to Pilo + Sham animals (Figure 1A). The same pattern was observed after 5 days of stimulation (DBS main effect *F*_(1,12)_ = 297.90, *p* < 0.0001; Figure 1B), with the additional observation that mean adenosine levels in the DBS + Pilo group were almost twice as high as their own values on stimulation day 1 (*p* < 0.001). In both days 1 and 5, adenosine remained increased for at least 1 h following DBS discontinuation. Levels of this nucleoside were significantly higher in chronic epileptic animals as compared to non-epileptic controls (Pilo main effect *F*_(1,12)_ = 114.88, *p* < 0.001).

### Antiepileptic effects of AN DBS *in vitro*

We have previously found that animals receiving DBS for 5 days had a significant decrease in hippocampal excitability measured *in vitro* with electrophysiology (Covolan et al., 2014). In preclinical models, the antiepileptic effects of adenosine occur largely through A1 receptors (Simonato et al., 1994; Ekonomou et al., 1998; Gouder et al., 2003; Rebola et al., 2003; Avsar and Empson, 2004; Mohammad-Zadeh et al., 2005, 2009; Fedele et al., 2006; Rosim et al., 2011; Silva et al., 2011). Based on these premises, we hypothesized that the increased adenosine levels recorded after DBS might be interacting with A1 receptors to reduce hippocampal excitability. To test this hypothesis, we recorded activity from slices of rats previously given 5 days of stimulation with or without co-administration of the A1 antagonist 8-Cyclopentyl-1,3-dipropylxanthine (DPCPX) or the A1 agonist R-isomer of N6-phenylisopropyladenosine (R-Pia). In these experiments, we did not record *in vivo* seizure rate due to the marked variability observed across animals in our previous report (Covolan et al., 2014).

**Figure 2:**
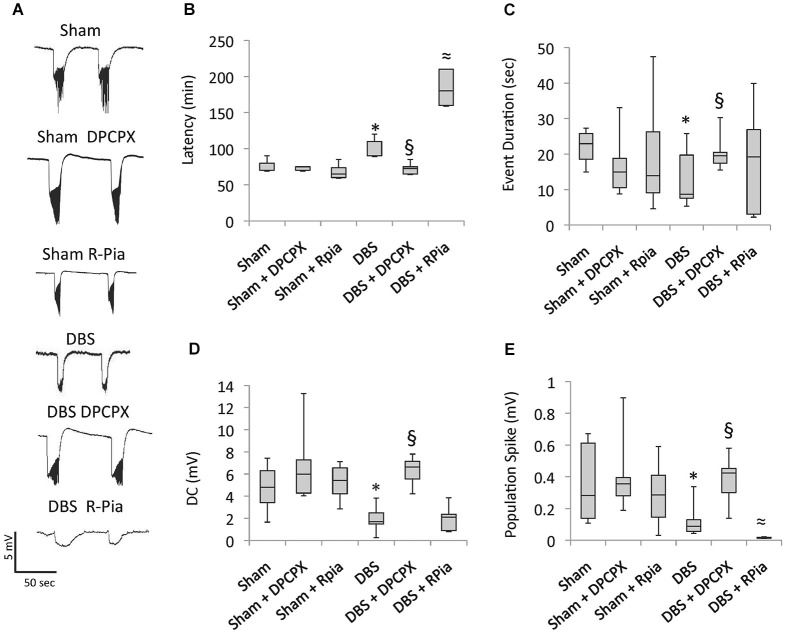
**Antiepileptic effects of AN DBS are mediated by A1 receptors**. Chronic epileptic rats undergoing AN stimulation were concomitantly given either the A1 receptor antagonist 8-cyclopentyl-1,3-dipropylxanthine (DPCPX) or the A1 agonist R-N6-(2)-phenylisopropyladenosine (R-Pia). **(A)** *In vitro* recordings under a zero calcium high potassium condition show epileptiform discharges characterized by DC shifts intermingled with spiking activity. **(B–E)** Box-and-whisker plots show that animals previously given DBS at 100 µA had a longer latency for the development of epileptiform activity, shorter and smaller DC shifts, and a smaller spike amplitude as compared to slices from animals previously given no stimulation. The decrease in hippocampal excitability observed after AN stimulation was almost completely reversed in slices from animals previously given DBS + DPCPX. R-Pia potentiated the antiepileptic effects of DBS. The box shows median, quartile 1 and 3. The whiskers show minimal and maximal values. Significant differences between the groups (*p* < 0.05; Kruskall Wallis followed by Mann Withney) are represented as follows: DBS vs. sham *; DBS vs. DBS + DPCPX §; and DBS vs. DBS + R-Pia ≈.

Confirming our previous findings (Covolan et al., 2014), epileptiform activity in slices of animals previously given AN DBS had longer latencies (*p* = 0.04), with shorter (*p* < 0.01) and smaller direct current (DC) shifts (*p* < 0.01), and a smaller spike amplitude (*p* < 0.01), as compared to non-stimulated epileptic controls (Figure 2). These effects were completely abolished in animals given DPCPX (Figure 2). In contrast, the protective effects of DBS were somewhat potentiated in animals given DBS plus R-Pia. Slices from this group had lower spike amplitudes (*p* < 0.01) and a longer latency for the development of epileptiform events (*p* < 0.01) as compared to those from rats receiving DBS alone (Figure 2). No differences in activity have been recorded in slices from animals given saline, DPCPX or R-Pia alone.

## Discussion

Reduced hippocampal excitability and susceptibility for the development of ictal activity after AN DBS in slices from chronic epileptic animals were completely blocked by treatment with A1 antagonists and potentiated by the co-administration of the A1 agonist R-Pia. This suggests that hippocampal adenosine released after AN stimulation may activate A1 receptors and subsequently lead to structural and/or functional changes capable of rendering the hippocampus less susceptible to ictal events.

The first interesting aspect of our study was the long-lasting stimulation-induced release of adenosine. This nucleoside has been shown to inhibit seizure activity both *in vitro* and in animal models of epilepsy (Dunwiddie, 1980; Van Dycke et al., 2010). We found that in chronic epileptic animals, the increase in adenosine release after DBS not only outlasted the administration of current but was significantly more pronounced at long-term. Bearing in mind the antiepileptic effects of adenosine, one may speculate that the frequency of seizures and epileptiform activity after AN stimulation could become less pronounced with time. Though this has not been demonstrated in animals, it has been reported in clinical trials (Fisher et al., 2010). In a recent randomized controlled multicenter study, the effects of AN DBS have been shown to build up with time, being far more pronounced at long-term (Fisher et al., 2010). Our results obviously do not allow us to conclude that adenosine is involved in the increased efficacy of DBS over time but they do suggest a path to be explored in future studies.

Due to the high variability in seizure rate observed in the pilocarpine model, we did not record the frequency of behavioral or electrographic seizures in our dialysis experiments. That said, animals selected for that part of the study were not those with high number of seizures at baseline, as we were afraid of them damaging the dialysis set up while having generalized ictal events (this in fact did happen with one of our rats). A few animals in the epileptic groups did have seizures that lasted a few seconds during the experiments. As our samples were collected over 30 min, associated changes in levels of adenosine could not be captured. Another interesting finding in our study was the fact that epileptic animals had higher baseline levels of adenosine in the hippocampus compared to non-epileptic controls. Whether this is a compensatory mechanism to hinder seizure activity remains to be investigated. Finally, one may argue that the simple implantation of electrodes in the brain might have contributed to our results. We find this unlikely for two main reasons. First, electrodes were implanted a few millimeters away from the collection site (AN vs. hippocampus). Second, no differences in baseline adenosine levels have been recorded between DBS treated animals and their respective controls (i.e., prior to stimulation onset).

We have focused on A1 receptors as these are highly expressed in the hippocampus (Swanson et al., 1995) and have been extensively demonstrated to be involved in the inhibition of ictal activity (Fedele et al., 2006). Overall, our electrophysiological experiments have shown that (1) the decrease in hippocampal excitability observed after AN stimulation was almost completely reversed in slices from animals previously given A1 antagonists; and (2) previous administration of A1 agonists to animals receiving DBS potentiated the *in vitro* antiepileptic effects of stimulation. Our results suggest that adenosine released after DBS would activate A1 receptors and induce functional and/or structural changes that might have decreased hippocampal excitability and the propensity for seizures. In our study, electrophysiology results in slices from animals receiving R-Pia alone were not identical to those recorded in the DBS group. This may be due to the fact that the effects of DBS are fairly complex, involving synaptic and non-synaptic mechanisms as well as multiple neurotransmitter systems. A more suggestive finding supporting the role of A1 receptors was the reduced hippocampal excitability recorded in animals previously given DBS was completely abolished by the co-administration of DPCPX.

As a final remark we would like to point out two caveats of our study. First, we have only recorded electrophysiological data 5 days after DBS onset, an interval that was based on our previous report (Covolan et al., 2014). In contrast, our dialysis experiments were conducted at two time points. As in our previous studies DBS was shown to induce an increase in neurotransmitter release right after stimulation onset (Hamani et al., 2010), we have decided to record adenosine levels within the first hours of DBS administration. To understand whether changes in adenosine levels after stimulation were sustained and still noticeable within the timeframe of our electrophysiological recordings, we have also conducted dialysis experiments after 5 days of DBS. At present, it is still unclear whether the administration of A1 antagonists blocks the effects of 1-day DBS treatment. As a second remark, we did not record behavioral seizure rate, dialysis samples and *in vitro* electrophysiology in the same animals for the reasons described above. As a result, we could not correlate these events and are unable to provide a clear causal relationship between hippocampal excitability, adenosine levels and the frequency of behavioral seizures.

In summary, we found that the purinergic system may be involved in the antiepileptic effects of AN DBS. In the clinical scenario, 40–60% of patients with treatment refractory epilepsy are responsive to AN DBS (Fisher et al., 2010). Therapeutic strategies for individuals considered as being non-responders remain unclear. Though no pure A1 agonist is available for clinical use, our findings may pave the way for future investigation on the use of medications that influence A1 receptors to potentiate the effects of stimulation.

## Materials and methods

Protocols were approved by the Animal Care committee of the Universidade Federal de São Paulo (CEUA 1482/11).

### Surgery and an stimulation

Four months after pilocarpine injections (Pilo; 320 mg/Kg i.p.), adult male Wistar rats (250–300 g) were divided in groups to receive DBS or sham-surgery (holes drilled in the skull). Animals with implanted electrodes that did not receive stimulation have not been included as such treatment did not lead to significant differences as compared to non-implanted controls in our previous work (Covolan et al., 2014). Under ketamine/xylazine anesthesia (100/7.5 mg/kg i.p.), animals in the DBS group had insulated stainless steel electrodes (cathodes; 250 µm diameter; 0.5 mm exposed length) bilaterally implanted into the AN (anteroposterior −1.5 mm, lateral ±1.5 mm, depth 5.2 mm) (Paxinos and Watson, 1998). A screw implanted over the right somatosensory cortex was used as the anode. On the second postoperative week, DBS was administered for 5 days (8 h/day) using a portable stimulator (St Jude Medical, Plano, TX) at 130 Hz, 90 µs, and 100 µA. These settings were chosen as they were effective against pilocarpine-induced chronic epileptic seizures in our previous study (Covolan et al., 2014).

### Electrophysiology

Prior to the experiments, chronic epileptic rats were given 5 days of DBS with or without the concomitant administration of the A1 receptor antagonist DPCPX (50 µg/kg/day i.p.) or agonist R-Pia (25 µg/kg/day i.p.) (Vianna et al., 2005). Following CO_2_ narcosis, animals were decapitated, brains were removed and 400 µm hippocampal slices were cut on a vibratome. Slices were then individually transferred to an interface-type chamber, placed on a membrane (0.4 µm Millicell culture plate inserts; Millipore, Badford, MA) and continuously bathed with artificial cerebrospinal fluid (aCSF; 127 mM NaCl, 2 mM KCl, 1.5 mM MgSO_4_, 1.1 mM KH_2_PO_4_, 26 mM NaHCO_3_, 2 mM CaCl_2_, and 10 mM glucose) at 33°C under a stream of moisturized 95% O_2_—5% CO_2_. Slices were left undisturbed for 40 min before being perfused with a zero calcium and 8 mM potassium solution for approximately 1 h and 30 min (de Almeida et al., 2008).

Extracellular field potentials were recorded from the hippocampal dentate gyrus (DG), as previously described (de Almeida et al., 2008). For each group, slices containing the dorsal hippocampus of six different animals were analyzed (14 extracellular potentials per animal). The following parameters were studied: latency for epileptiform discharges after perfusion with a zero calcium high potassium solution; DC shift; amplitude of population spikes; and event duration. A digital Fourier transform was used to quantify DC shifts. Once in the frequency domain, the event signal was recalculated taking into account only components below 10 Hz. This process allowed the analysis of DC shifts without interference from population spikes. Event duration was calculated by subtracting the final time from the initial time of an event (de Almeida et al., 2008).

### Microdialysis

Along with the implantation of DBS electrodes, a microdialysis cannula was placed into the right dorsal hippocampus (anteroposterior −3.7 mm, lateral 2.4 mm, depth 5.1 mm) (Paxinos and Watson, 1998). Five days later, a microdialysis probe (CMA/12-2 mm, CMA Microdialysis, Sweden) was inserted into the target and perfused with a Ringer’s solution at 1 µL/min (Macedo et al., 2012). Dialysate samples were collected every 30 min in vials containing 0.25 mM ascorbic acid, Na_2_EDTA 0.27 mM, 0.1 M acetic acid on the first and last days of treatment at baseline (2 h), during DBS (3 h), and in the 3 h following stimulation offset (*n* = 4 animals per group).

Adenosine levels were detected with capillary electrophoresis (Beckman-Coulter PACE/MDQ, Beckman Coulter, USA) equipped with a laser-induced fluorescence detector (ZETALIF Picometrics, France; He-Cd laser wavelength 410 nm). Dialysate samples (20 µl) were derivatizated with 20 µl of a chloroacetaldehyde solution (0.15 M) (Sigma, USA). This mixture was allowed to react at 100°C for 20 min, subsequently refrigerated at 4°C and then injected into the capillary system. Adenosine analysis was carried out using a 20 mM sodium-phosphate buffer (containing 1% SDS, pH 8). The applied voltage was 20 kV. Samples were hydrodynamically injected (0.4 nl) in a fused-silica capillary (Beckman-Coulter, 25 µm i.d. and 375 µm o.d., 60 cm in length, and an effective length of 40 cm). The capillary was sequentially flushed with sodium hydroxide (0.1 M), ultra-pure water, and running buffer between each analysis. Electropherograms were acquired at 15 Hz using a PACE MDQ software (Beckman-Coulter).

### Statistical analyses and histology

Dialysis results for baseline, DBS and post-DBS periods were each analyzed with a mixed model ANOVA, with pilocarpine and DBS as between-subject factors and time as a within-subjects factor. As electrophysiological data was non-parametric, results were compared using the Kruskall Wallis and Mann Whitney tests. Dialysis data is displayed as mean ± standard error. Electrophysiology results are shown as median ± quartiles. Statistical significance was considered when *p* ≤ 0.05.

## Conflict of interest statement

Clement Hamani is a consultant for St Jude Medical. The other authors do not have a conflict of interest related to this article.
